# Ischemic and haemorrhagic stroke risk estimation using a machine-learning-based retinal image analysis

**DOI:** 10.3389/fneur.2022.916966

**Published:** 2022-08-22

**Authors:** Yimin Qu, Yuanyuan Zhuo, Jack Lee, Xingxian Huang, Zhuoxin Yang, Haibo Yu, Jinwen Zhang, Weiqu Yuan, Jiaman Wu, David Owens, Benny Zee

**Affiliations:** ^1^Centre for Clinical Research and Biostatistics, Jockey Club School of Public Health and Primary Care, The Chinese University of Hong Kong, Hong Kong, China; ^2^Centre for Clinical Trials and Biostatistics Lab, CUHK Shenzhen Research Institute, Shenzhen, China; ^3^School of Population Medicine and Public Health, Chinese Academy of Medical Science (CAMS) and Peking Union Medical College (PUMC), Beijing, China; ^4^Shenzhen Traditional Chinese Medicine Hospital, Shenzhen, China; ^5^Affiliated Shenzhen Maternity and Child Healthcare Hospital, Southern Medical University, Shenzhen, China; ^6^Swansea University, Wales, United Kingdom

**Keywords:** stroke subtypes classification, retinal image analysis, ischemic stroke, haemorrhagic stroke, machine-learning method

## Abstract

**Background:**

Stroke is the second leading cause of death worldwide, causing a considerable disease burden. Ischemic stroke is more frequent, but haemorrhagic stroke is responsible for more deaths. The clinical management and treatment are different, and it is advantageous to classify their risk as early as possible for disease prevention. Furthermore, retinal characteristics have been associated with stroke and can be used for stroke risk estimation. This study investigated machine learning approaches to retinal images for risk estimation and classification of ischemic and haemorrhagic stroke.

**Study design:**

A case-control study was conducted in the Shenzhen Traditional Chinese Medicine Hospital. According to the computerized tomography scan (CT) or magnetic resonance imaging (MRI) results, stroke patients were classified as either ischemic or hemorrhage stroke. In addition, a control group was formed using non-stroke patients from the hospital and healthy individuals from the community. Baseline demographic and medical information was collected from participants' hospital medical records. Retinal images of both eyes of each participant were taken within 2 weeks of admission. Classification models using a machine-learning approach were developed. A 10-fold cross-validation method was used to validate the results.

**Results:**

711 patients were included, with 145 ischemic stroke patients, 86 haemorrhagic stroke patients, and 480 controls. Based on 10-fold cross-validation, the ischemic stroke risk estimation has a sensitivity and a specificity of 91.0% and 94.8%, respectively. The area under the ROC curve for ischemic stroke is 0.929 (95% CI 0.900 to 0.958). The haemorrhagic stroke risk estimation has a sensitivity and a specificity of 93.0% and 97.1%, respectively. The area under the ROC curve is 0.951 (95% CI 0.918 to 0.983).

**Conclusion:**

A fast and fully automatic method can be used for stroke subtype risk assessment and classification based on fundus photographs alone.

## Background

Stroke is one of the most important causes of morbidity and mortality worldwide. It is the second leading cause of death, accounting for 6.3 million deaths in 2015 worldwide (1). Despite the decreasing trend in China for stroke prevalence since the 1990s, the absolute number of deaths and the loss of disability-adjusted life-years keep increasing (2). Stroke has become the leading cause of mortality (3), with an age-standardized mortality rate of 114.8/100 000 person-years in 2013 (4). The overall stroke burden is exceptionally high in rural areas where medical resources are limited (4).

As therapeutic options are limited, especially in rural areas, feasible and effective screening strategies are needed to identify high-risk stroke patients. Traditional methods to assess stroke risk include ultrasound, computed tomography angiography (CTA), and magnetic resonance angiography (MRA). Ultrasound can evaluate vascular stenosis and assess blood flow velocity in the carotid artery. Yet, some research has reported that carotid stenosis is not a good enough tool for stroke screening since most stroke patients do not have moderate or high stenosis that could have been detected before an incidence of stroke (5, 6). CTA and MRA can detect extensive cerebrovascular abnormalities (7). These techniques are valid with high accuracy, yet the relatively high cost and invasive quality made them impossible to be used as screening tools. Recently, there are digital solutions to assess stroke risk for the purpose of prevention (8). However, these tools were derived from the Framingham Stroke Risk Score prediction algorithm and were enhanced to include additional lifestyle risk factors shown to be important for stroke and CVD occurrence. The additional factors may be a helpful indication of risk or a response to the outcome. The advantage is that the algorithm is easy to use but the accuracy remains a question. More study is needed to find better factors to raise the predictive accuracy. Therefore, there is an urgent need for additional techniques to detect the subtle changes, ideally at an early stage before the incidence occurs, so that prevention can be considered to avoid the damage.

Retinal vessels are the only visible vessels accessible by simple fundus photography (9). They have the same embryo origin and histological structure as cerebral vessels (10–13). Retinal microvascular damages can reflect damage to cerebral microvasculature and neurons (14). It provides us with a convenient way to assess cardiovascular conditions. Previous studies have demonstrated that retinal characteristics contain valuable information for stroke risk assessment and conventional clinical variables (15–20). In addition, retinal microvasculature may provide adequate information to explain the underlying pathophysiological changes of various stroke subtypes (21).

In addition to finding indicators to establish a model for stroke risk estimation, identifying stroke subtypes is also vital for guiding clinical treatment and management. Ischemic stroke is due to a lack of blood flow and accounts for about 80% of strokes. Haemorrhagic stroke is due to bleeding and accounts for about 20% of strokes (22). Stroke subtyping can have different purposes. First, classifying patients is needed for therapeutic decision-making in clinical practice. An ischemic stroke may be treatable with a medication that can break down the clot, such as aspirin. While a haemorrhagic stroke may benefit from surgery (23). Haemorrhagic stroke has a much higher death rate than ischemic stroke (24). The strategies for preventing haemorrhagic and ischemic stroke are similar but not the same due to different disease pathology (25). Ischemic stroke prevention requires a comprehensive approach to the variety of stroke risk factors a patient may encounter. Similarly, prevention for haemorrhagic stroke will have to target efforts against the vascular risk factors significant in the haemorrhage's etiology. For preventing ischemic stroke, platelet antiaggregant and anticoagulant medications are usually required. In contrast, some degree of avoidance of these same medications is an issue in preventing haemorrhagic stroke (25).

This study aimed to establish risk estimation models for ischemic and haemorrhagic stroke patients and contribute to the early classification of the two-stroke subtypes with retinal characteristics.

## Study subjects

The stroke cases for this study were obtained from the Shenzhen Traditional Chinese Medicine (SZTCM) Hospital. Cases were defined as ischemic stroke patients and haemorrhagic stroke patients. Control subjects included patients with hypertension, dyslipidemia, or diabetes at the same hospital. In addition, we have increased the number of control subjects with healthy subjects from the community. The participants' inclusion criteria included good health status and ability to sit on a chair for retinal image taking; having clear basic information and disease diagnosis; having appropriate blood pressure measures, blood glucose, and blood lipids. Subjects with any of the following conditions were excluded from the study: cataracts or other eye diseases that affected retinal image taking, requiring close clinical monitoring, being too weak to comply with the research, and being unable or unwilling to comply with disease examination. For this study, we have obtained clinical research ethics approval from the Shenzhen Traditional Chinese Medicine Hospital Ethics Committee (Ref. No.: K2019-005-01) and the Joint Chinese University of Hong Kong—New Territories East Cluster Clinical Research Ethics Committee (Ref. No.: 2020.093). Baseline information, including age, sex, smoking, drinking, and medical history, was collected by trained doctors upon admission. Trained nurses measured patients' height, weight, and blood pressure on the first day of admission. Body mass index (BMI) was calculated as the weight (kg) divided by the square of the height (m). The blood samples were collected in the morning. A fully automatic analyser measured blood lipid levels of total cholesterol (TC), triglycerides (TG), high-density lipoprotein (HDL) cholesterol, low-density lipoprotein (LDL) cholesterol, fasting blood glucose level, glycated hemoglobin test (HbA1c), coagulation index levels of activated partial thromboplastin time (APTT), prothrombin time (PT), fibrinogen (FIB), thrombin time (TT.); and blood routine examination results including white blood cell (WBC), hemoglobin, blood platelet (PLT), and plateletcrit (PCT). Hypertension was defined as a systolic blood pressure of ≥ 140 mmHg or diastolic blood pressure of ≥ 90 mmHg or hypertension history. Diabetes mellitus was defined as fasting plasma glucose ≥ 6.1 mmol/L or HbA1c ≥ 5.8% or having a diabetes history. Dyslipidemia was defined as total cholesterol (TC) ≥ 240 mg/L, triglyceride (TG) ≥ 200 mg/dL, HDL <40 mg/dL, low-density lipoprotein cholesterol (LDL-c) ≥ 160 mg/dL, or as patients with a history of dyslipidemia. All patients underwent detailed radiographic evaluations, including a cranial magnetic resonance imaging (MRI) scan and a duplex color Doppler ultrasound or contrast-enhanced cranial magnetic resonance imaging angiography (MRA) (26). In addition, retinal photography was taken within 2 weeks of hospital admission.

## Methods

Quantitative variables were expressed as the mean ± standard deviation, and categorical variables were expressed as counts with percentages. For univariate analysis, independent *t*-tests were conducted to compare continuous data between groups, and the chi-square tests were conducted for categorical data analysis. A fully automatic retinal image analysis for stroke subtypes was developed using R and Matlab computer software to estimate retinal microvascular characteristics and incorporate machine-learning techniques to estimate risks of ischemic and haemorrhagic strokes. The detailed methods of the automatic retinal imaging analysis method have been reported previously for studies related to cerebral magnetic resonance imaging (27–29).

The odds ratios (OR) and 95% confidence interval (CI) were reported for variables in the model. To ensure the consistency of the models and to avoid overfitting, we have conducted a 10-fold cross-validation analysis. The sensitivity, specificity, and area under the receiver operating characteristic curve (AUC of ROC) were reported for each model. The Delong method was used to compare the difference between AUCs (30). *P* < 0.05 was considered as statistical significance.

For the classification models, we used machine-learning and deep learning techniques. Using Matlab, we first applied a transfer net “ResNet50” convolutional neural network with retinal images as input. The outputs were features generated based on pixels associated with stroke subtype status. We also extracted the texture/fractal/spectrum-related features (such as high order spectra and fractal dimensions) associated with stroke subtypes using the automatic retinal image analysis (ARIA) algorithm written in Matlab (31). We then used the glmnet approach to select the most important subset of features based on the penalized maximum likelihood using R and Matlab. These refined features are highly associated with stroke subtypes. Finally, we translated the features extracted from the above machine-learning approaches to commonly used retinal characteristics measured from the images using ImageJ. This part of the analysis, performed with SPSS, helped enhance our understanding of retinal characteristics that contribute to the classification and identification of specific stroke subtypes.

### Retinal parameters estimation

Canon non–mydriatic retinal camera (Canon-CR2) was used to capture the retinal color image using a 45° field of view centered on the fovea. The retinal characteristics measurement tools previously developed as part of the ARIA algorithm were used to estimate the parameters of retinal vessels. The retinal characteristics measurement previously developed as part of the Automatic Retinal Image Analysis (ARIA) algorithm was used to estimate the parameters of retinal vessels (31, 32). The following is a brief description:

**Retinal vessel measurements**: Our estimates were based on the formula developed by Knudtson et al. (33) to describe the retinal vessel measurements into the central retinal artery equivalent (CRAE) and central retinal vein equivalent (CRVE). **Arteriole-venous nipping and arteriole occlusion**: The sign of arteriole-venous nipping was marked as the narrowing of the venule at the crossing point of arteriole. The arteriole occlusions (Aocclusion) were presented as thread-like arterioles when the blood inside the arterioles was stopped by emboli. **Hemorrhages and exudates**: Status of hemorrhages and exudates were recorded as either present or absent. Hemorrhages and exudates were key determinants for the severity of diabetic retinopathy as they were found to be associated with stroke in other studies. **Tortuosity**: Tortuosity was assessed by visual grading of one fovea-centered and one disc-centered fundus image from each image. The grading levels for retinal arterial tortuosity were either predominantly straight arteries or mild to severe tortuosity with at least one inflection of at least one major artery. **Bifurcation coefficients (BC)**: Bifurcation coefficient (BC) is the ratio of the sum of the cross-sectional areas of the daughter vessels of a bifurcation to that of the parent stem. The means of the bifurcation coefficient of arterioles (BCA) and venules (BCV) were used. **Asymmetry of branches and bifurcation angles**: Asymmetry index (AI) is the ratio of diameters of two daughter branches. The AI was calculated as AI=D1/D2, where D1 and D2 were smaller and larger branches, respectively. The mean of the three sets of AI of arterioles (Aasymmetry) and venules (Vasymmetry) was used. The angle between two daughter branches of the same branches studied in the BC was measured. The centerline of two branches was drawn, and the angle was calculated to represent the branching angle. The mean of the bifurcation angles of arterioles (Aangle), and mean of bifurcation angles of venules (Vangle) from the three sets of vessels in one retinal image were used for the analysis.

### Clinical risk factors estimation

In addition to the retinal microvascular characteristics, we used machine-learning techniques to estimate important clinical risk factors and distinguish stroke subtypes. Previous studies reported several clinical characteristics differences between haemorrhagic stroke and ischemic stroke (34). For example, Zhang et al. (35) reported that ischemic stroke patients are significantly older (*p* < 0.001), have a higher proportion of family history of stroke (*p* = 0.01), obesity (*p* < 0.001), diabetes (*p* = 0.004), TIA (*p* = 0.017), atrial fibrillation (*p* = 0.002), lower level of HDL (*p* = 0.001), and carotid atheroma (*p* = 0.002). At the same time, haemorrhagic stroke patients have a higher proportion of males (*p* = 0.023), alcohol drinking (*p* = 0.003), hypertension (*p* = 0.003), and increased WBC (*p* < 0.001). Our study would use retinal images to estimate the clinical risk factors and compare the control, haemorrhagic, and ischemic stroke groups to provide further insight into the retinal image analysis for stroke subtypes classification.

## Results

Seven hundred eleven patients were enrolled, including 145 ischemic stroke patients, 86 haemorrhagic patients, and 480 controls. Among the 480 controls, 123 came from the Shenzhen TCM Hospital and 357 from healthy volunteers in the community. Descriptive statistics for stroke subtype (ischemic stroke / haemorrhagic stroke) and control related to baseline information and cardiovascular risk factors are shown in Table 1. For the comparison between ischemic stroke and control groups, age, systolic and diastolic blood pressure, and hypertension were significantly higher, but the proportion of males was significantly smaller. The same pattern occurred for the haemorrhagic stroke compared to the control group, except that significantly more males were in the haemorrhagic stroke group.

**Table 1:** Comparison of clinical characteristics between control, ischemic and hemorrhagic stroke.

	**Control**	**Ischemic stroke**	**Hemorrhagic stroke**
	**(*N* = 480)**	**(*n* = 145)**	**(*N* = 86)**
Age, mean (95% CI)^1^	42.74 (41.52–43.96)	58.90 (57.08–60.73) *	52.12 (49.63–54.60) *
BMI, mean (95% CI)^1^	24.01 (23.68–24.34)	23.58(23.02–24.15)	23.68 (22.97–24.39)
SBP, mean (95% CI)^1^	123.05 (121.42–124.68)	131.04(128.06–134.02) *	131.27 (131.09–136.29) *
DBP, mean (95% CI)^1^	75.53 (74.35–76.71)	80.85(78.82–82.87) *	82.02 (79.12–84.93) *
Male, *N* (%)^2^	244 (50.8%)	118(81.4%) *	71 (80.6%) *
Hypertension, *N* (%)^2^	83 (56.5%)	122 (84.1%) *	80 (93.0%) *
Smoker, *N* (%)^2^	50 (10.4%)	19(8.4%)	9 (10.5%)

^1^*Independent sample t test*.

*^2^Chi-square test.*

^*^P <0.001 compared to control.

For the retinal characteristics, CRAE and CRVE, AVR, and bifurcation coefficients were significantly smaller in both the ischemic and haemorrhagic stroke groups. The other retinal characteristics such as bifurcation angles, asymmetry, tortuosity, nipping, hemorrhages, occlusion, and exudates have significantly larger values in the stroke-subtype groups than in control (Tables 2, 3). These results show many differences in retinal characteristics among the control group and the ischemic and haemorrhagic stroke groups.

**Table 2:** Comparison of retinal characteristics between control and ischemic stroke.

**Characteristics**	**Control**	**Ischemic**	***P* value**
	**(*N* = 480)**	**(*N* = 145)**	
**Retinal characteristics**			
lCRAE	13.155 (13.052–13.258)	11.336 (11.279–11.393)	<0.001
lCRVE	20.240 (20.127–20.354)	18.239 (18.167–18.311)	<0.001
lMBCA	1.626 (1.618–1.634)	1.613 (1.604–1.623)	0.056
lMBCV	1.308 (1.302–1.314)	1.215 (1.209–1.220)	<0.001
lMVangle	70.903 (70.678-71.128)	73.936 (73.672–74.199)	<0.001
lMAangle	74.062 (73.877-74.247)	75.249 (74.966–75.532)	<0.001
lMAasymmetry	0.826 (0.825–0.828)	0.847 (0.845–0.850)	<0.001
lMVasymmetry	0.771 (0.769–0.773)	0.742 (0.740–0.745)	<0.001
lTortuosity	0.264 (0.256–0.271)	0.293 (0.283–0.304)	<0.001
lNipping	0.250 (0.239–0.261)	0.310 (0.300–0.320)	<0.001
lHemorrhage	0.254 (0.241–0.267)	0.303 (0.285–0.321)	<0.001
lAocclusion	0.097 (0.089–0.105)	0.137 (0.121–0.153)	<0.001
lExudates	0.170 (0.161–0.180)	0.247 (0.234–0.260)	<0.001
lAVR	0.649 (0.647–0.651)	0.622 (0.619–0.624)	<0.001
rCRAE	12.853 (12.753–12.953)	11.104 (11.060–11.147)	<0.001
rCRVE	19.958 (19.850–20.065)	18.176 (18.109–18.243)	<0.001
rMBCA	1.600 (1.592–1.608)	1.606 (1.596–1.617)	0.345
rMBCV	1.286 (1.281–1.291)	1.199 (1.195–1.203)	<0.001
rMVangle	71.677 (71.419–71.936)	75.008 (74.710–75.306)	<0.001
rMAangle	73.402 (73.154–73.649)	76.370 (76.101–76.639)	<0.001
rMAasymmetry	0.834 (0.833–0.836)	0.842 (0.840–0.845)	<0.001
rMVasymmetry	0.778 (0.776–0.779)	0.756 (0.754–0.758)	<0.001
rTortuosity	0.302 (0.296–0.309)	0.329 (0.317–0.341)	<0.001
rNipping	0.272 (0.265–0.279)	0.320 (0.309–0.331)	<0.001
rHemorrhage	0.254 (0.245–0.263)	0.353 (0.335–0.370)	<0.001
rAocclusion	0.071 (0.066–0.076)	0.114 (0.097–0.132)	<0.001
rExudates	0.138 (0.130–0.146)	0.240 (0.226–0.254)	<0.001
rAVR	0.643 (0.641–0.645)	0.611 (0.609–0.614)	<0.001

**Table 3:** Comparison of retinal characteristics between control and hemorrhagic stroke.

**Characteristics**	**Control**	**Hemorrhagic stroke**	***P* Value**
	**(*N* = 480)**	**(*N* = 86)**	
**Retinal characteristics**			
lCRAE	13.155 (13.052–13.258)	11.325 (11.257–11.393)	<0.001
lCRVE	20.240 (20.127–20.354)	18.227 (18.142–18.311)	<0.001
lMBCA	1.626 (1.618–1.634)	1.622 (1.608–1.636)	0.730
lMBCV	1.308 (1.302–1.314)	1.212 (1.205–1.220)	<0.001
lMVangle	70.903 (70.678–71.128)	73.798 (73.425–74.170)	<0.001
lMAangle	74.062 (73.877–74.247)	75.299 (74.899–75.698)	<0.001
lMAasymmetry	0.826 (0.825–0.828)	0.848 (0.845–0.851)	<0.001
lMVasymmetry	0.771 (0.769–0.773)	0.746 (0.742–0.749)	<0.001
lTortuosity	0.264 (0.256–0.271)	0.290 (0.275–0.305)	0.001
lNipping	0.250 (0.239–0.261)	0.308 (0.293–0.323)	<0.001
lHemorrhage	0.254 (0.241–0.267)	0.300 (0.279-0.322)	<0.001
lAocclusion	0.097 (0.089–0.105)	0.136 (0.113–0.159)	<0.001
lExudates	0.170 (0.161–0.180)	0.237 (0.220–0.254)	<0.001
lAVR	0.649 (0.647–0.651)	0.622 (0.618–0.625)	<0.001
rCRAE	12.853 (12.753–12.953)	11.131 (11.069–11.193)	<0.001
rCRVE	19.958 (19.850–20.065)	18.227 (18.125–18.329)	<0.001
rMBCA	1.600 (1.592–1.608)	1.606 (1.592–1.621)	0.540
rMBCV	1.286 (1.281–1.291)	1.200 (1.194–1.206)	<0.001
rMVangle	71.677 (71.419–71.936)	75.349 (74.964–75.734)	<0.001
rMAangle	73.402 (73.154–73.649)	76.453 (76.112–76.794)	<0.001
rMAasymmetry	0.834 (0.833–0.836)	0.841 (0.838–0.843)	<0.001
rMVasymmetry	0.778 (0.776–0.779)	0.757 (0.755–0.760)	<0.001
rTortuosity	0.302 (0.296–0.309)	0.328 (0.312–0.344)	0.003
rNipping	0.272 (0.265–0.279)	0.323 (0.308–0.337)	<0.001
rHemorrhage	0.254 (0.245–0.263)	0.359 (0.335–0.383)	<0.001
rAocclusion	0.071 (0.066–0.076)	0.116 (0.096–0.136)	<0.001
rExudates	0.138 (0.130–0.146)	0.229 (0.212–0.247)	<0.001
rAVR	0.643 (0.641–0.645)	0.611 (0.608–0.614)	<0.001

Table 4 shows the risk factors for control, ischemic stroke and haemorrhagic stroke. Comparing the three groups concerning clinical characteristics estimated from retinal images is to demonstrate that the retinal images contain information for the classification of stroke subtypes based on known significant clinical variables. In our study, we found that ischemic stroke patients who are older (*p* = 0.001) have more diabetes (*p* < 0.001) and carotid atherosclerosis (*p* < 0.001) than haemorrhagic stroke. In addition, both haemorrhagic and ischemic strokes have significantly more males, a higher proportion of patients with hypertension, atrial fibrillation (AF), lacunar infarct, and carotid atherosclerosis.

**Table 4:** The distribution of estimated Clinical risk factors for the three classes of control, Ischemic stroke and Hemorrhagic Stroke.

**Clinical factors**	**Control**	**Hstroke**	**Istroke**	***P***	***P1***	***P2***	***P3***
Gender (Male)	35.0%	84.9%	81.4%	< .001	< .001	< .001	.496
Hypertension (Yes)	61.8%	90.7%	87.6%	< .001	< .001	< .001	.469
DM (Yes)	18.7%	18.6%	46.9%	< .001	.986	< .001	< .001
Current smoking (Yes)	9.8%	9.3%	11.7%	0.804	0.913	0.605	.567
Current drinking (Yes)	12.2%	8.1%	6.9%	0.304	0.347	0.137	0.727
CHD (Yes)	0.0%	3.5%	10.3%	< .001	0.037	< .001	0.060
AF (Yes)	0.0%	7.0%	8.3%	0.006	0.003	0.001	0.722
Hyperlipidemia (Yes)	66.7%	60.5%	64.1%	0.655	0.358	0.665	0.577
Lacunar infarction (Yes)	0.0%	33.7%	31.7%	< .001	< .001	< .001	0.754
Carotid atherosclerosis (0 vs. 1, 2)	48.8%	74.4%	91.7%	< .001	< .001	< .001	< .001
Age (Mean)	51.44	53.76	58.75	< .001	0.260	< .001	0.001
BMI (Mean)	24.60	23.29	24.11	0.003	0.004	0.356	0.090

*P is the p-value for the chi-square test comparing all three groups; P1 is the p-value for comparison between the control group and Hstroke (hemorrhagic stroke) group, P2 is the p-value for comparison between the control group and Istroke (ischemic stroke) group, P3 for comparison between Hstroke group and Istroke group*.

**Figure 1:**
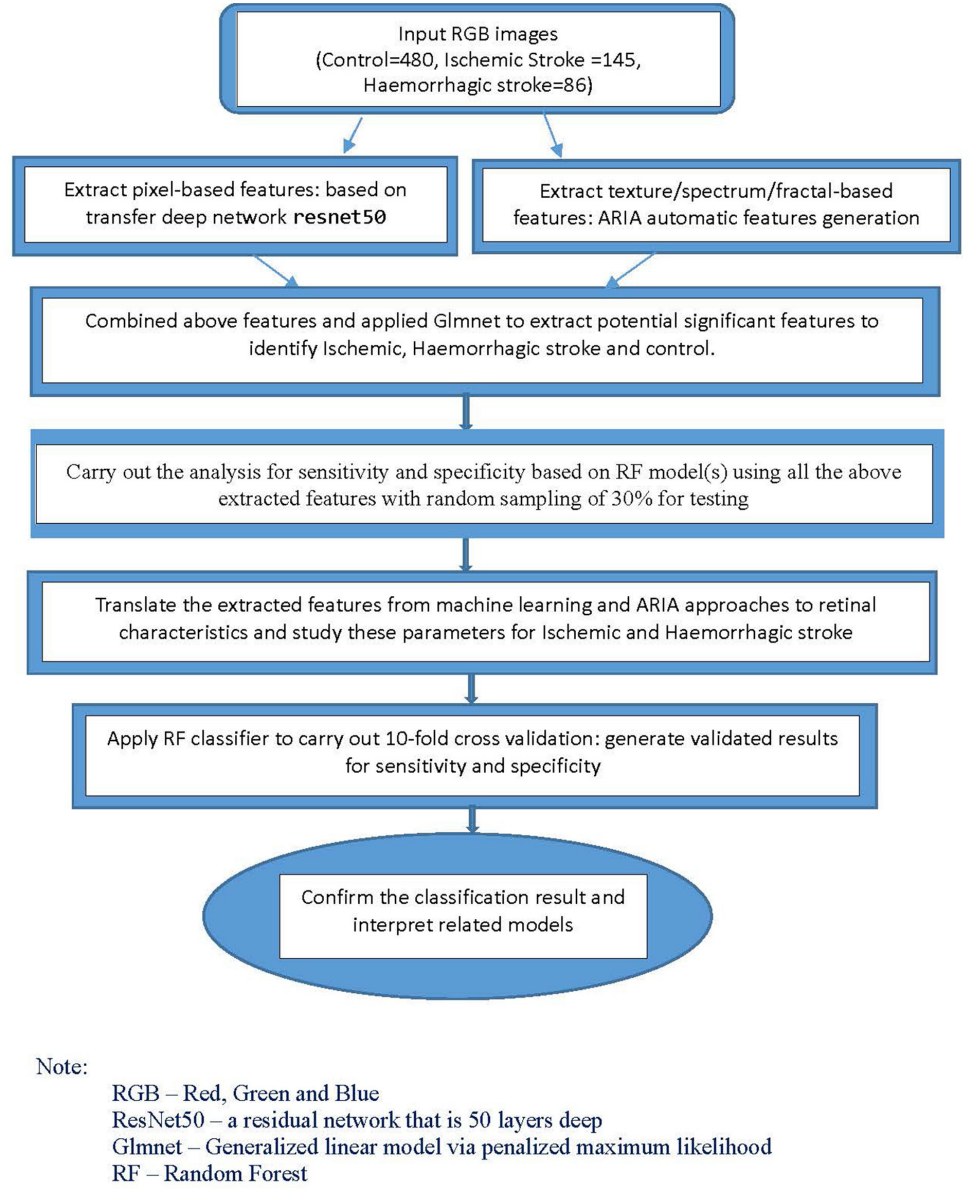
Flowchart of the method for the development of the classification model.

**Figure 2:**
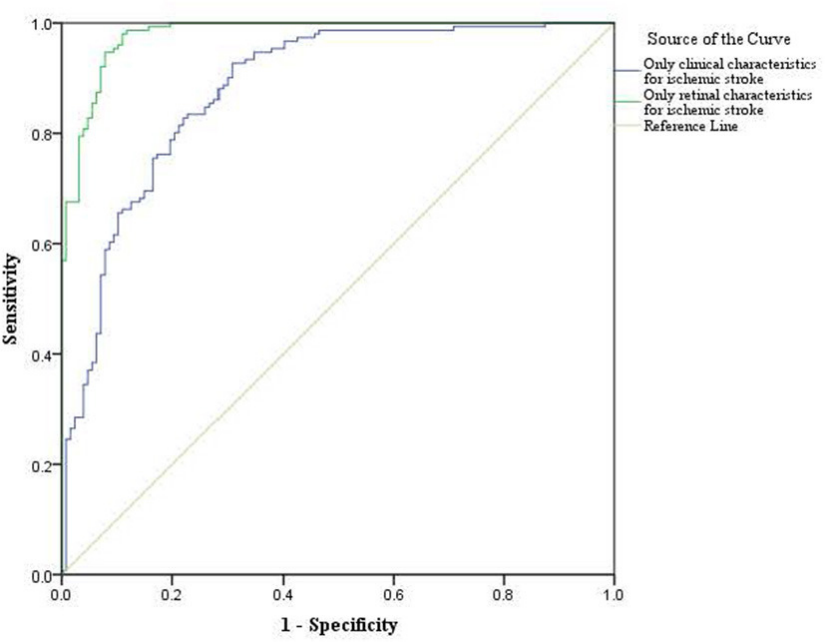
ROC curves of the model using only clinical characterisitics and model using only retinal characteristics for ischemic stroke risk estimation.

**Figure 3:**
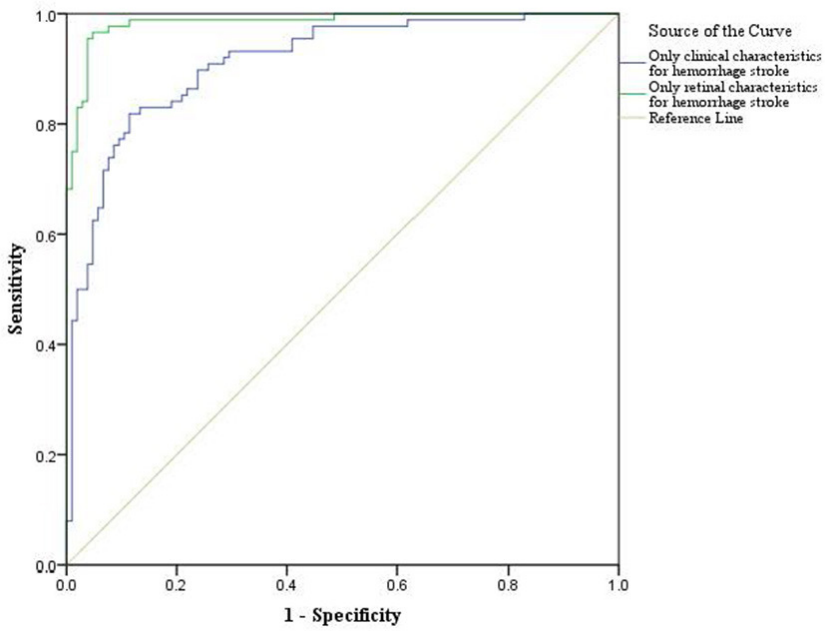
ROC curves of the model using only clinical characteristics and model using only retinal characterisitcs for haemorrhagic stroke risk estimation.

For the classification analysis, Figure 1 shows the flow chart for the methods. We have analyzed the classification performance between using retinal characteristics alone vs. clinical characteristics using logistic regression. Delong's method was used to compare the AUCs of models. The results show that retinal characteristics performed significantly better than clinical characteristics alone (*p* < 0.001). The AUC for ischemic stroke based on clinical and retinal variables was 0.88 (95% CI of 0.84, 0.92) and 0.98 (95% CI of 0.97, 0.99), respectively (Figure 2). The AUC for haemorrhagic stroke based on clinical and retinal variables were 0.91(95% CI 0.87, 0.95) and 0.98 (95% CI of 0.97, 1.00), respectively (Figure 3).

**Figure 4:**
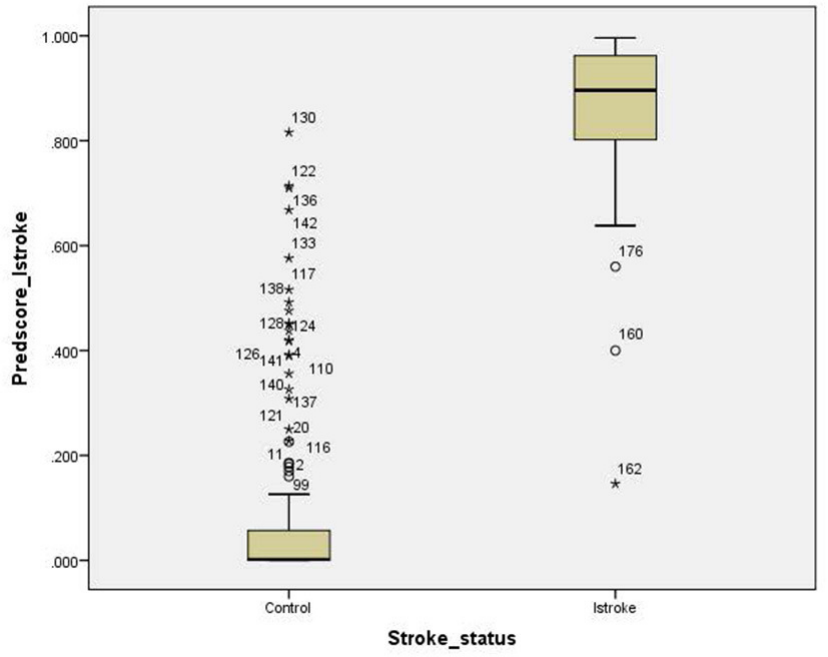
Box plot for the probability of ischemic stroke.

**Figure 5:**
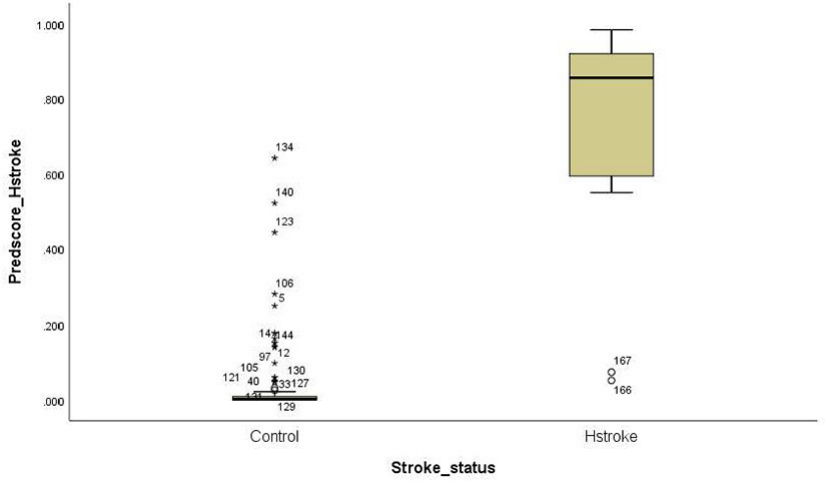
Box plot for the probability of haemorrhagic stroke.

**Figure 6:**
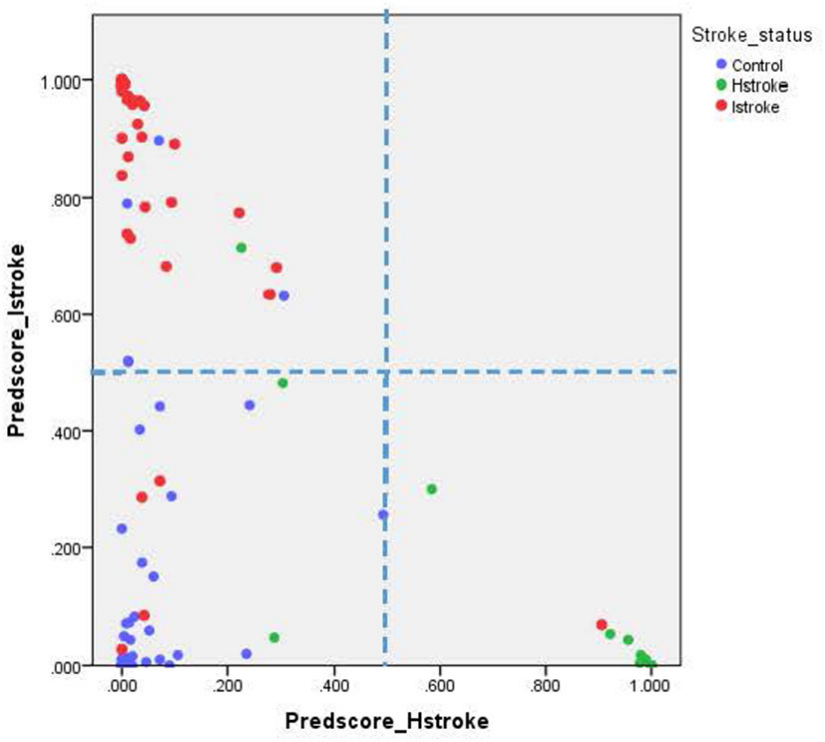
Scatter plot for both ischemic and haemorrhagic strokes.

For the ischemic stroke classification model, the 10-fold cross-validation gives sensitivity and specificity of 91.0% and 94.8%, respectively. The area under the ROC for ischemic stroke based on the 10-fold cross-validation analysis was 0.929 (95% CI of 0.900–0.958). The box plot for the probability of ischemic stroke is shown in Figure 4. For haemorrhagic stroke, the sensitivity and specificity were 93.0% and 97.1%, respectively. The area under the ROC for haemorrhagic stroke based on the 10-fold cross-validation was 0.951 (95% CI of 0.918–0.983). The box plot for the probability of haemorrhagic stroke is shown in Figure 5. Since there is an age difference between the control and the stroke groups, we carried out further investigation. Among the 480 controls in this study, 123 came from the same SZTCM hospital and 357 from healthy volunteers in the community. The average age of the 123 controls from SZTCM hospital was 52.13, similar to the haemorrhagic stroke patients. If we only use these 123 controls as the control group, the risk estimation models also perform well. The classification model for ischemic stroke vs. control had a sensitivity of 90.63%, a specificity of 91.56%, and an AUC of 0.98. The classification model for haemorrhagic stroke vs. control had a sensitivity of 92.97%, a specificity of 85.56%, and an AUC of 0.98. This result demonstrated the robustness of the models regardless of age. Both the sensitivity and the specificity of the two-stroke subtypes have high accuracy. Still, it is also essential to know if they can discriminate the two-stroke subtypes. We demonstrated these classification models for stroke subtypes have good discrimination power using the 30% validation portion of the data. In the scatter-plot, the classification models discriminate the probability of ischemic stroke, haemorrhagic stroke and control subjects in three apparent clusters, as shown in Figure 6. The figure can be divided into four quadrants if we draw a horizontal line for the ischemic stroke (y-axis) and a vertical line for haemorrhagic stroke (x-axis). The ischemic stroke patients were clustered in the top left-hand quadrant, with a high probability of ischemic stroke but a low probability of haemorrhagic stroke. The haemorrhagic stroke patients clustered in the lower right-hand quadrant, with a high probability of haemorrhagic stroke but a low probability of ischemic stroke. The control subjects clustered around the origin in the lower left-hand quadrant, with a low probability for both ischemic and haemorrhagic strokes.

When we evaluated the stroke subtypes together, the error rates for all three groups are shown in Table 5. For ischemic stroke patients, 9/145 (6.2%) were misclassified to control, and 3/145 (2.1%) were misclassified to haemorrhagic stroke. For haemorrhagic stroke, 4/86 (4.7%) were misclassified to control, and 11/86 (12.8%) were misclassified to ischemic stroke. For the control, the error rate was 29/480 (6%), with 28/480 (5.8%) misclassified as ischemic stroke and 1/480 (0.2%) misclassified as haemorrhagic stroke.

**Table 5:** Ten-fold cross-validation of all three groups.

**Results of classification**	**Control**	**Ischemic stroke**	**Hemorrhagic stroke**
	**(*n* = 480)**	**(*n* = 145)**	**(*n* = 86)**
Control	451	9	4
Ischemic stroke	28	133	11
Hemorrhagic stroke	1	3	71
**% Correct**	**94%**	**91.7%**	**82.6%**

## Discussion

The performance of the risk estimation models for ischemic and haemorrhagic strokes was excellent. We have a sensitivity and specificity of 91.0% and 94.8% for ischemic stroke classification and 93.0% and 97.1% for haemorrhagic stroke classification, respectively. These results showed that retinal characteristics were highly efficient in classifying stroke subtypes. In addition, we can now evaluate both risks of stroke subtypes longitudinally to study how they are related during their development.

The retinal characteristics are of significant interest as markers of stroke since they can be directly visualized *via* ophthalmoscopy (27). Previous studies have shown that retinal vascular changes vary according to stroke subtypes (17, 36–38). Clinical risk factors provide general associations for stroke risk estimation, but they do not classify ischemic and haemorrhagic stroke separately. However, stroke prevention and clinical management rely on accurate risk estimation and classification. Many routine preventive treatments for ischemic stroke, including antiplatelet therapy, anticoagulants, and statins, have been noted to generate a higher risk for haemorrhagic stroke. For example, aspirin for platelet therapy would increase haemorrhagic stroke risk (39–42). Study on the use of clopidogrel for antiaggregant yielded a similar result (43). Anticoagulation such as warfarin for stroke prevention also increases the risk of intracerebral hemorrhage (34, 44–46). As a result, knowing the risk of stroke subtypes at an early stage is highly advantageous.

We further employed the machine-learning method to estimate clinical risk factors using retinal images and showed the differences between control, ischemic stroke, and haemorrhagic stroke agree with previous literature for stroke subtypes based on clinical risk factors. This analysis demonstrated that the retinal image contains information on the clinical variables that contributed to the model classification (35). Other investigations using retinal image information for clinical application are starting to appear. For example, a recent study shows that multifractals of the retinal vessel can be used to predict pial collateral status for patients with ischemic stroke (47).

Finally, we can use the estimated stroke subtypes risks as a target for designing a health and wellness plan from a prevention point of view. Prevention trials or lifestyle intervention studies are now feasible with the retinal image analysis approach.

### Limitations

There are several limitations to this study. First, we did not have a separate data set for model validation in this research. Thus, we have conducted the 10-fold cross-validation, and the results showed that the performance of the models was stable on the training data set and the cross-validation data set. Second, the sample size was relatively small, which may affect the statistical power of the classification. Third, the haemorrhagic stroke cases in this study are mainly intracerebral hemorrhages. We have no subarachnoid hemorrhage sample in this study. Therefore, the classification may not apply to subarachnoid haemorrhagic stroke cases. Finally, since this is a case-control study, we cannot establish the temporal relationship if the retinal changes before the onset of the stroke.

### Future direction

This study is a pioneer study with a potential future clinical application where we can apply the results from retinal imaging to the hospital Accident and Emergency (A&E) Department as a screening tool for stroke risk including subtypes classification. The management of ischemic and hemorrhagic strokes is very different. Retinal imaging is fast and convenient, it will provide crucial information for the A&E operation and help prioritize patients' specific needs for CT or MRI confirmation in a timely fashion.

## Data availability statement

The raw data supporting the conclusions of this article will be made available by the authors, without undue reservation.

## Ethics statement

The studies involving human participants were reviewed and approved by Joint CUHK-NTEC Clinical Research Ethics Committee. The patients/participants provided their written informed consent to participate in this study.

## Author contributions

YQ and BZ contributed to the study design and writing of the initial draft of the manuscript. YQ, JZ, and JW contributed to the data collection. YZ, ZY, and HY contributed to data collection and their interpretation. DO and JW provided clinical advice and interpretation. JL and BZ carried out methodological development and retinal image analysis. JL and YQ provided statistical analysis and prepared figures and tables. All authors reviewed the manuscript, made significant contributions, and approved the submitted version.

## Funding

This study was supported by the General Research Fund (GRF) of the Hong Kong Research Grant Council (No. 14139116); National Natural Science Foundation of China (No. 81803952); Science Technology and Innovation Commission of Shenzhen Municipality (KCXFZ20201221173208024).

## Conflict of interest

Authors BZ and JL have a patent “Method and device for retinal image analysis” licensed to Health View Bioanalytic Limited and received royalties through the Chinese University of Hong Kong. BZ and JL are founders and shareholders of Health View Bioanalytic Limited, Bioanalytic Holdings Limited, and Bioanalytic International Holdings Limited. The remaining authors declare that the research was conducted in the absence of any commercial or financial relationships that could be construed as a potential conflict of interest.

## Publisher's note

All claims expressed in this article are solely those of the authors and do not necessarily represent those of their affiliated organizations, or those of the publisher, the editors and the reviewers. Any product that may be evaluated in this article, or claim that may be made by its manufacturer, is not guaranteed or endorsed by the publisher.
