# Corrigendum: Ischemic and haemorrhagic stroke risk estimation using a machine-learning-based retinal image analysis

**DOI:** 10.3389/fneur.2023.1256958

**Published:** 2023-07-27

**Authors:** Yimin Qu, Yuanyuan Zhuo, Jack Lee, Xingxian Huang, Zhuoxin Yang, Haibo Yu, Jinwen Zhang, Weiqu Yuan, Jiaman Wu, David Owens, Benny Zee

**Affiliations:** ^1^Centre for Clinical Research and Biostatistics, Jockey Club School of Public Health and Primary Care, The Chinese University of Hong Kong, Hong Kong, China; ^2^Centre for Clinical Trials and Biostatistics Lab, CUHK Shenzhen Research Institute, Shenzhen, China; ^3^School of Population Medicine and Public Health, Chinese Academy of Medical Science (CAMS) and Peking Union Medical College (PUMC), Beijing, China; ^4^Shenzhen Traditional Chinese Medicine Hospital, Shenzhen, China; ^5^Affiliated Shenzhen Maternity and Child Healthcare Hospital, Southern Medical University, Shenzhen, China; ^6^Swansea University, Wales, United Kingdom

**Keywords:** stroke subtypes classification, retinal image analysis, ischemic stroke, haemorrhagic stroke, machine-learning method

In the published article, there was an error in the order of the affiliations. Instead of “^1^Centre for Clinical Research and Biostatistics, Jockey Club School of Public Health and Primary Care, The Chinese University of Hong Kong, Hong Kong, China, ^2^Centre for Clinical Trials and Biostatistics Lab, CUHK Shenzhen Research Institute, Shenzhen, China, ^3^Shenzhen Traditional Chinese Medicine Hospital, Shenzhen, China, ^4^Affiliated Shenzhen Maternity and Child Healthcare Hospital, Southern Medical University, Shenzhen, China, ^5^Swansea University, Wales, United Kingdom, ^6^School of Population Medicine and Public Health, Chinese Academy of Medical Science (CAMS) and Peking Union Medical College (PUMC), Beijing, China”, it should be “^1^Centre for Clinical Research and Biostatistics, Jockey Club School of Public Health and Primary Care, The Chinese University of Hong Kong, Hong Kong, China, ^2^Centre for Clinical Trials and Biostatistics Lab, CUHK Shenzhen Research Institute, Shenzhen, China, ^3^School of Population Medicine and Public Health, Chinese Academy of Medical Science (CAMS) and Peking Union Medical College (PUMC), Beijing, China, ^4^Shenzhen Traditional Chinese Medicine Hospital, Shenzhen, China, ^5^Affiliated Shenzhen Maternity and Child Healthcare Hospital, Southern Medical University, Shenzhen, China, ^6^Swansea University, Wales, United Kingdom”.

The authors apologize for this error and state that this does not change the scientific conclusions of the article in any way. The original article has been updated.

